# More evidence of Impact Factor Mania

**DOI:** 10.1128/spectrum.03496-23

**Published:** 2023-11-01

**Authors:** Patrick D. Schloss, Christina A. Cuomo

**Affiliations:** 1 Department of Microbiology and Immunology, University of Michigan, Ann Arbor, Michigan, USA; 2 Department of Molecular Microbiology and Immunology, Brown University, Providence, Rhode Island, USA

## EDITORIAL

*Microbiology Spectrum* was initially launched in 2011 to provide authors who were publishing monographs with a PubMed indexed, citable publication. Because ASM made the strategic decision over the past five years to move away from publishing a large volume of monographs, the pipeline for monographs to populate *Microbiology Spectrum* immediately began to dry up. The last monograph-based issue of *Microbiology Spectrum* was published in December 2020. At the same time, the ASM Journals Committee appreciated that many authors, including ASM members, were leaving ASM’s journals after receiving a rejection and then publishing their work elsewhere. Our assessment was that, in many cases, the papers were sound but perceived by reviewers and editors as being of limited impact or novelty. Regardless, considering so many papers were leaving the ASM’s Journals Program, it was clear that we were not adequately serving the needs of the ASM community. Rather than retire *Microbiology Spectrum* and create another journal title, we saw an opportunity to rebrand *Microbiology Spectrum* as a "sound science" journal that emphasized the scientific rigor rather than the potential impact or significance of the manuscript. The new *Microbiology Spectrum* was launched in June 2021 under Christina Cuomo, PhD, as its new Editor in Chief (1). Despite being only a little over 2 years old, by prioritizing an open process for joining the editorial board, Dr. Cuomo and the journal team have developed an amazing, international, and very diverse community of over 450 editors. The journal has also developed a mentoring program that allows early career scientists to be mentored into Editorial roles. We believe that the journal will continue to thrive because of its commitment not only to sound science but also to diversity, equity, and inclusion in the editorial and peer review process.

As we worked to rebrand the journal as an open access research title, there was a special interest in openness of data, publishing negative and confirmatory results, and publishing work that was perceived to be sound. This included studies too specific to a subfield or geographic region to be as impactful as the work published in the other ASM journals. There has also been an emphasis on recruiting editors and associate editors to reflect the broad geographic, gender, racial, and scientific diversity of ASM. Papers published in *Microbiology Spectrum* are accepted by transferring manuscripts rejected from other ASM journals with their peer review history or by *de novo* submissions. From the outset, *Microbiology Spectrum* decided that it would not accept review articles because we wanted to focus on the mission of publishing sound science. Overall, we expected that without the requirement for novelty or potential impact of articles, most manuscripts published at *Microbiology Spectrum* may not garner a large number of citations. However, published papers would serve as a knowledge seed bank to provide a starting point for future disease outbreaks from previously poorly studied pathogens (e.g., Zika, SARS) and as a resource to help reduce the “file drawer effect” common in scientific publishing (2). We were amazed by the initial excitement to publish in the rebranded *Microbiology Spectrum*. We received thousands of submissions in 2022 and orders of magnitude higher than originally anticipated at launch.

As we relaunched *Microbiology Spectrum* with a large marketing effort and launched the first substantial issue of new content with an editorial (1), we expected authors to be aware of the radical change in the scope of the journal. Two years into the relaunch of *Microbiology Spectrum*, we realize that we inadvertently performed a natural experiment of researchers’ use of journal impact factors. A journal’s impact factor is the average number of citations papers received in a year divided by the number of citable items the journal published in the two preceding years. This number is closely watched by some publishers, funding agencies, and universities. Because of the significant effects publishing in a journal with a large impact factor can have, authors often are particularly aware of a journal’s impact factor. The obsessive use of journal impact factors to evaluate individual people’s research based on where they publish has previously been referred to as "Impact Factor Mania" (3).

In 2012, the San Francisco Declaration on Research Assessment (DORA) was written to stop the practice of attaching a measure of a journal’s impact with an individual scientist’s contributions (4). As a signatory of DORA, ASM does not publicize its journals impact factors (5). Although some take this to mean that ASM’s journals do not have impact factors, they do. Each year, Clarivate calculates and publicizes the impact factors for each of ASM’s journals regardless of ASM’s desires.

ASM’s reasons for signing the declaration and not publicizing impact factors are numerous. A few examples highlight the problems. First, the metric is not scientific. For example, it ignores the problem with calculating a mean rather than a median using data with a skewed distribution. It also ignores the significant number of papers that are never cited or that are only cited after a several year delay. It is also a metric that is easy to skew. For example, review articles are highly cited and so publishing more reviews can boost a journal’s impact factor without changing the quality or impact of the research they are publishing. Also, editorials and commentaries such as this are not counted as a citable item, but citations to them do count. So, if a reader cites this editorial, it will count to the numerator but not the denominator boosting *Microbiology Spectrum*’s impact factor. Thus, journals are incentivized to publish more of this material to boost their impact factors. Second, it is biased toward journals that publish studies in popular areas and garner more citations (e.g., clinically oriented journals), which are not necessarily the most important areas. Ten years ago, Zika virus and SARS were not seen as being impactful. Yet, ASM felt it was important to publish those papers to more fully represent the microbial sciences. Had we been concerned with optimizing impact factors, those papers would not have been published and our responses to those outbreaks would have been slower. Third, it allows journals to be far more selective in what they publish and how many papers they publish. Each year, a journal can publish 100 carefully selected papers from highly cited areas and garner a much higher impact factor than a journal that publishes 1,000 high-quality papers from a diverse swath of areas. We contend that the latter journal does a better job of serving the members of ASM and science in general.

With these critiques of journal impact factors, we hope that it is clear why we relaunched *Microbiology Spectrum* without regard for its impact factor. By publishing papers that were specialized, confirmatory, or presented negative results, we expected fewer citations per paper than we would have seen at ASM’s other journals. By not publishing review articles, editorials, or commentaries, *Microbiology Spectrum* could not be accused of trying to game their impact factor. In July 2022, the journal’s final impact factor from the monograph-based scope was 9.043 (https://journalimpact.org/). This was calculated using 2021 citations for the content published in 2019 and 2020. Now, in June 2023, using four papers published with the last year of the old scope and a year of papers with the new scope, the impact factor fell to 3.7. This is in the range of other journals publishing sound science articles, which will be the continued focus on *Microbiology Spectrum*.

We suspect that some authors were using the journal impact factors of ASM journals to determine where to submit their manuscripts. Between June 2021 and May 2022, 64% were transfers, and between June 2022 and May 2023, 44% of the papers the journal received were transfers from other ASM journals, and the remainder were *de novo* submissions. This higher proportion of direct submissions coincided with the period when *Microbiology Spectrum* had a higher impact factor. The drop in the 2022 impact factor had a significant impact on the number of submissions to the journal. Comparing the number of submissions in 2022 and 2023, the number of submissions to *Microbiology Spectrum* for the month of May increased by 12.1% and they decreased by 43.7% for July. These results of a natural experiment of relaunching a journal by radically changing its scope demonstrate a fundamental problem with impact factors and how scientists use them.

We are aware that a small fraction of *Microbiology Spectrum* authors feel that ASM did not do enough to maintain the journal’s impact factor. It is clear that these authors must not have considered the change in scope of the journal or the description of the journal’s mission (1), ASM’s marketing prior to submitting their work, or ASM’s resistance to impact factors (5). The quality of the science published in May 2023 is no different from that published in July 2023, but because the journal’s impact factor changed so drastically, there is a perception that the quality of the work has been diminished. One cannot simply distill the contents of a journal to a single number and we cannot assess the quality of a paper based on the science that it is published with. Scientists need to do a better job of assessing the quality and impact of a paper by reading it and critically evaluating the content. Alas, it appears that Impact Factor Mania has been a persistent infection.

Over the next several years, we expect the impact factor and reputation of *Microbiology Spectrum* to stabilize. Among the three open access, general scope journals that ASM publishes, we anticipate that *mBio*, *mSphere*, and *Microbiology Spectrum* will have impact factors that follow that order. We are proud of what *Microbiology Spectrum* stands for. Our improved ability to provide more comprehensive coverage of microbiology under an open access license with the support of a society-based publishing model will allow ASM to better serve its members by promoting and advancing the microbial sciences (6).

## AFTER EPUB

*[This article was published on 1 November 2023 with an error in the 2nd sentence. The sentence was corrected in the current version, posted on 6 November 2023.]*
